# The Homeobox Transcription Factor HOXA9 Is a Regulator of *SHOX* in U2OS Cells and Chicken Micromass Cultures

**DOI:** 10.1371/journal.pone.0045369

**Published:** 2012-09-20

**Authors:** Claudia Durand, Eva Decker, Ralph Roeth, Katja U. Schneider, Gudrun Rappold

**Affiliations:** Department of Human Molecular Genetics, University of Heidelberg, Heidelberg, Germany; University of Massachusetts Medical, United States of America

## Abstract

The homeobox gene *SHOX* encodes for a transcription factor that plays an important role during limb development. Mutations or deletions of *SHOX* in humans cause short stature in Turner, Langer and Leri-Weill syndrome as well as idiopathic short stature. During embryonic development, *SHOX* is expressed in a complex spatio-temporal pattern that requires the presence of specific regulatory mechanisms. Up to now, it was known that *SHOX* is regulated by two upstream promoters and several enhancers on either side of the gene, but no regulators have been identified that can activate or repress the transcription of *SHOX* by binding to these regulatory elements. We have now identified the homeodomain protein HOXA9 as a positive regulator of *SHOX* expression in U2OS cells. Using luciferase assays, chromatin immunoprecipitation and electrophoretic mobility shift assays, we could narrow down the HOXA9 binding site to two AT-rich sequences of 31 bp within the *SHOX* promoter 2. Virus-induced *Hoxa9* overexpression in a chicken micromass model validated the regulation of *Shox* by Hoxa9 (negative regulation). As *Hoxa9* and *Shox* are both expressed in overlapping regions of the developing limb buds, a regulatory relationship of Hoxa9 and Shox during the process of limb development can be assumed.

## Introduction

The human pseudoautosomal gene *SHOX* encodes for a homeodomain transcription factor with a crucial role during limb development and growth regulation [1], [2], [3]. Mutations or deletions of *SHOX* have been identified as the primary cause of several disorders characterized by reduced body height and skeletal deformities including the short stature associated with Turner Syndrome, Léri-Weill Dyschondrosteosis and Langer Syndrome [4], [5]. In these syndromes, the skeletal malformations manifest as a mesomelic shortening of the long bones of the limbs, particularly affecting the middle portion of the upper limbs, where a shortening and bowing of the radius leads to a dorsal subluxation of the ulna (Madelung deformity). The distinctly localized clinical symptoms are explained by the specific *SHOX* expression pattern in the developing limbs that is seen during embryonic and fetal development and later on during childhood. In human embryos, the most striking expression is seen in the middle part of the limb buds, where *SHOX* is initially expressed in the undifferentiated mesenchymal tissue. At later stages, when the mesenchyme condenses and endochondral ossification takes place, *SHOX* is mainly found in the perichondrial layer surrounding the forming bone [6]. Using immunohistochemical methods, the SHOX protein was also detected in the chondrocytes of fetal and childhood growth plates [7], [8]. These observations have implied a role of SHOX in bone development and offer an explanation for the localized symptoms seen in *SHOX*-deficient patients.

As *SHOX* is not existent in rodent genomes, developing chicken embryos present an important model system for the analysis of *SHOX* during limb development. In chicken, the *Shox* expression pattern corresponds very well to the expression in human embryos. In early stages, *Shox* is uniformly expressed in the central mesoderm of the limb bud. In later stages, expression is restricted to the proximal two thirds of the developing limb bud [9]. In human and chicken, *SHOX* expression in limb buds exhibits a small overlap with the expression of *SHOX2*, a highly related *SHOX* paralog. Whereas *SHOX* expression is restricted to the middle part of the limb bud in later stages, *SHOX2* expression is mainly seen in more proximal regions [6], [9].

**Figure 1:**
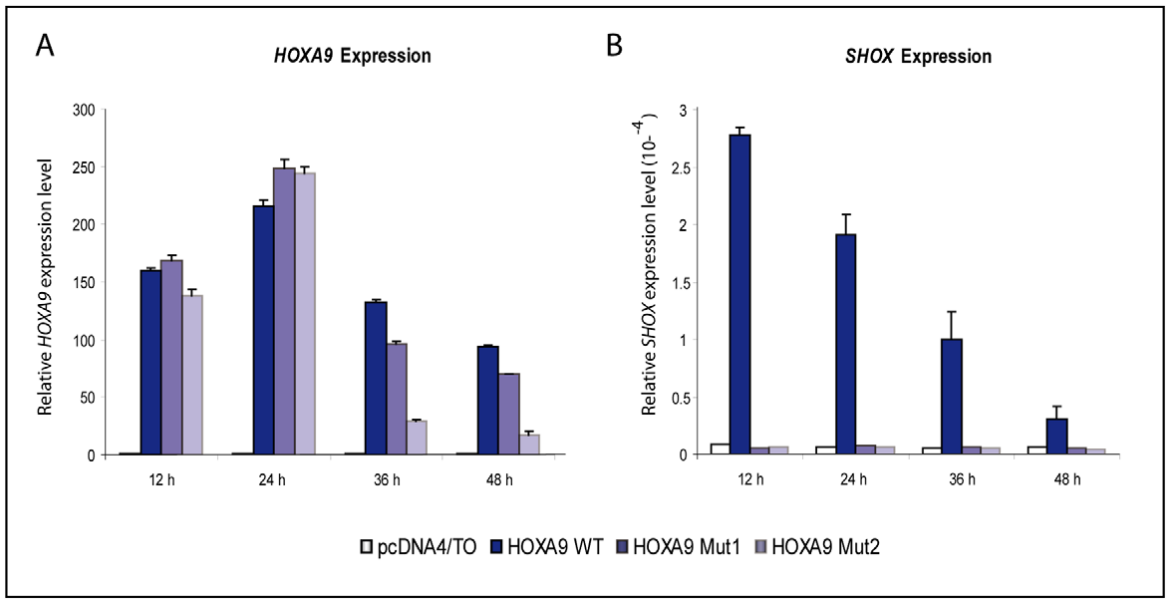
*HOXA9* overexpression in U2OS cells increases *SHOX* expression. (A) qRT-PCR analysis of *HOXA9* expression levels after transient overexpression of *HOXA9* in U2OS cells. A strong increase is seen upon transfection with wild type constructs as well as mutant constructs. (B) qRT-PCR analysis of *SHOX* expression levels after overexpression of *HOXA9*. *HOXA9* wild type, but not its mutants, is able to increase *SHOX* expression. *HOXA9 Mut1 = K223E; HOXA9 Mut2 = K223E, N256del, R257P, R258G.* All mutations affect highly conserved amino acids within the homeodomain.

The generation of such a distinct and restricted expression pattern as it is seen for *SHOX* requires specific regulatory input. In recent years, several cis-regulatory elements of *SHOX* have been discovered. *SHOX* expression is controlled by two alternative promoters that generate transcripts with identical coding capacity but different 5′UTRs leading to different translational efficiencies of the transcript [10]. In addition, enhancer elements residing up- and downstream of the gene were found to control *SHOX* expression [11], [12], [13], [14], [15]. However, the regulatory mechanisms that control *SHOX* and the molecular pathways in which it is involved during limb development remain elusive. So far, no regulators have been identified that activate or repress *SHOX* transcription by binding to its regulatory elements.

**Figure 2:**
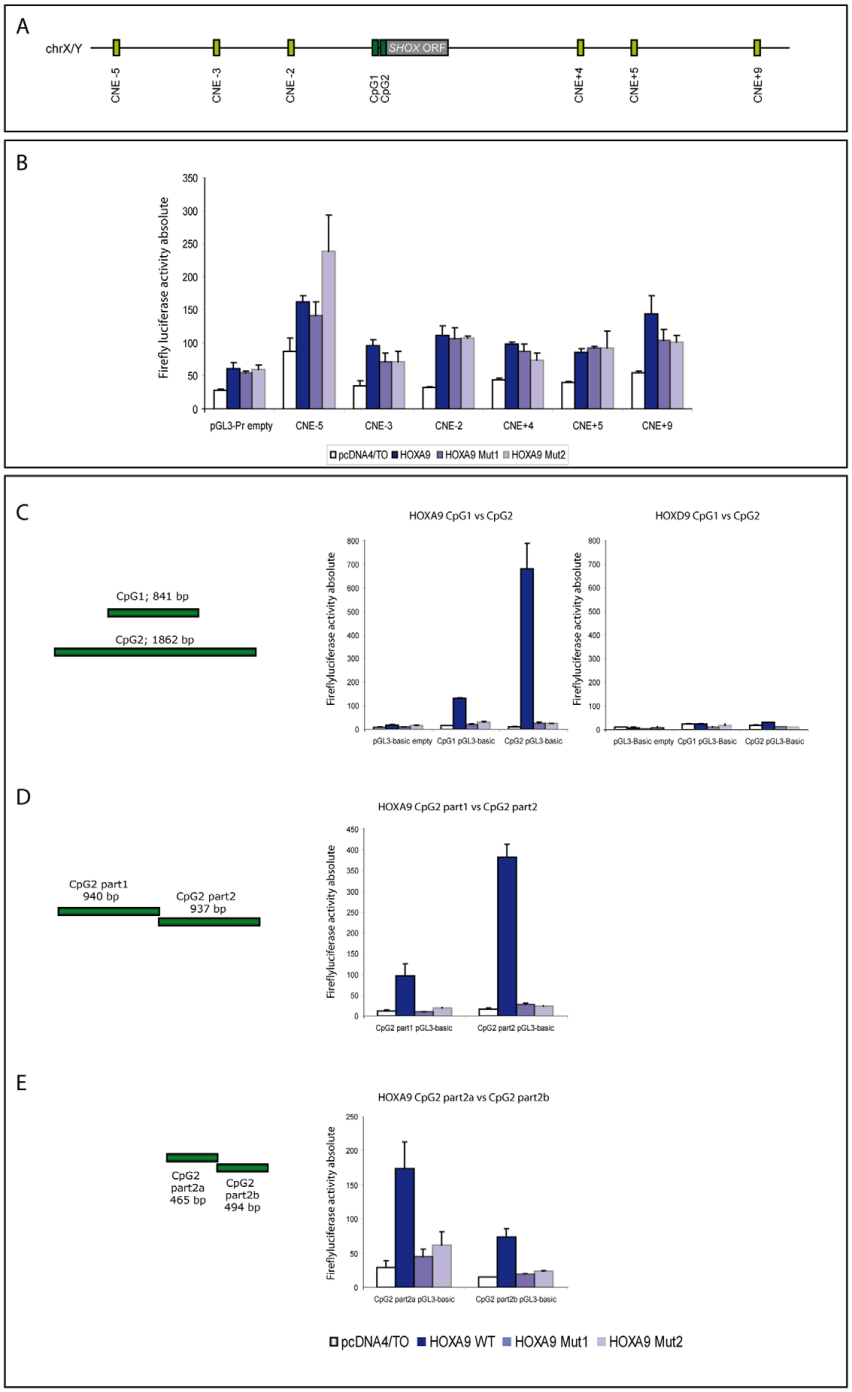
Luciferase assays with *SHOX* cis-regulatory elements. (A) Schematic overview of *SHOX* cis-regulatory elements (not drawn to scale). CpG 1 and 2, which contain the two *SHOX* promoters, encompass the regions of exon 1 and 2, respectively (CpG1 chrX/Y:504,564-505,326; CpG2 chrX/Y:510,430-512,197). In addition, there are six known limb specific enhancer elements (CNE-5: chrX/Y: 318,357-318,906; CNE-3 chrX/Y:380,279-380,664, CNE-2: chrX/Y:436,610-437,229; CNE4: chrX/Y:634,085-634,740; CNE5: chrX:670,705-671,956; CNE9: chrX:754,746+755,567). (B) Luciferase assays for the *SHOX* enhancers. *SHOX* enhancers were cloned upstream of a firefly luciferase into the vector pGL3-Promoter and cotransfected with a *HOXA9* expression vector or the empty or mutant control vectors, respectively. Overexpression of HOXA9 or its mutants produced only low increases of comparable levels of luciferase activity arguing for a HOXA9 independent effect. (C-D) Deletion analysis of *SHOX* CpG Islands 1 and 2 to narrow down the site of regulatory *HOXA9* activity by luciferase assays. (C) *SHOX* CpG Islands (schematically drawn as green bars) were cloned upstream a firefly luciferase into the vector pGL3-Basic and cotransfected with a *HOXA9* overexpression vector or the empty or mutant control vectors, respectively. Upon *HOXA9* expression, luciferase activity increases for CpG1 (8 fold) and for CpG2 (70 fold) (left and middle panel). As a control, CpG luciferase vectors were also cotransfected with *HOXD9* expression vectors and the respective control vectors. HOXD9 was not able to evoke an increase of luciferase activity as seen for HOXA9 (right panel). (D) Subdivision of CpG2 (as indicated by green bars). Upon *HOXA9* overexpression, a stronger increase of luciferase activity was seen for CpG2 part 2 than for part 1. (E) Subdivision of CpG2 part 2. CpG2 part 2a was able to evoke stronger luciferase activity compared with CpG2 part 2b. This region is therefore considered to inherit the main sites that are important for the HOXA9 mediated regulatory activity.

## Materials and Methods

### Generation of *HOX* Expression Constructs

Human *HOX* genes and the cofactors *PBX1* and *MEIS1* were amplified out of cDNA from U2OS cells (human osteosarcoma cells, ATCC) using a Flag-tagged reverse primer and were then cloned into the expression vector pcDNA4/TO (Invitrogen) via the multiple cloning site.

*In vitro* mutagenesis of the *HOXA9*- and *HOXD9* constructs was performed with the QuikChange Multi Site-Directed Mutagenesis Kit (Stratagene). All primers used are listed in Table S1.

### Cell Culture and Transient Transfection Assays

U2OS cells were cultured in DMEM (Dulbecco's Modified Eagle Medium; Gibco) containing 10% FBS (Fetal bovine serum Gold; PAA) and penicillin/streptomycin (Gibco) at 37°C, 5% CO_2_ and 95% humidity.

For overexpression experiments, 1–2×10^6^ cells were transfected with 1 µg of the respective Flag-tagged *HOX* expression constructs cloned into pcDNA4/TO. Transfections were carried out using either the Cell Line Nucleofector Kit V (Lonza) or Lipofectamine2000 Transfection reagent (Invitrogen) according to the manufacturer’s instructions. Medium was changed six hours after transfection.

### Preparation and Reverse Transcription of RNA

RNA from cell lines and chicken micromass cultures was prepared using the illustra RNA spin Mini Kit (GE Healthcare) according to the manufacturer’s protocol. Reverse transcription of 1 µg RNA was performed with Superscript III Reverse Transcriptase (Invitrogen) using random hexamer and oligo dT primers.

**Figure 3:**
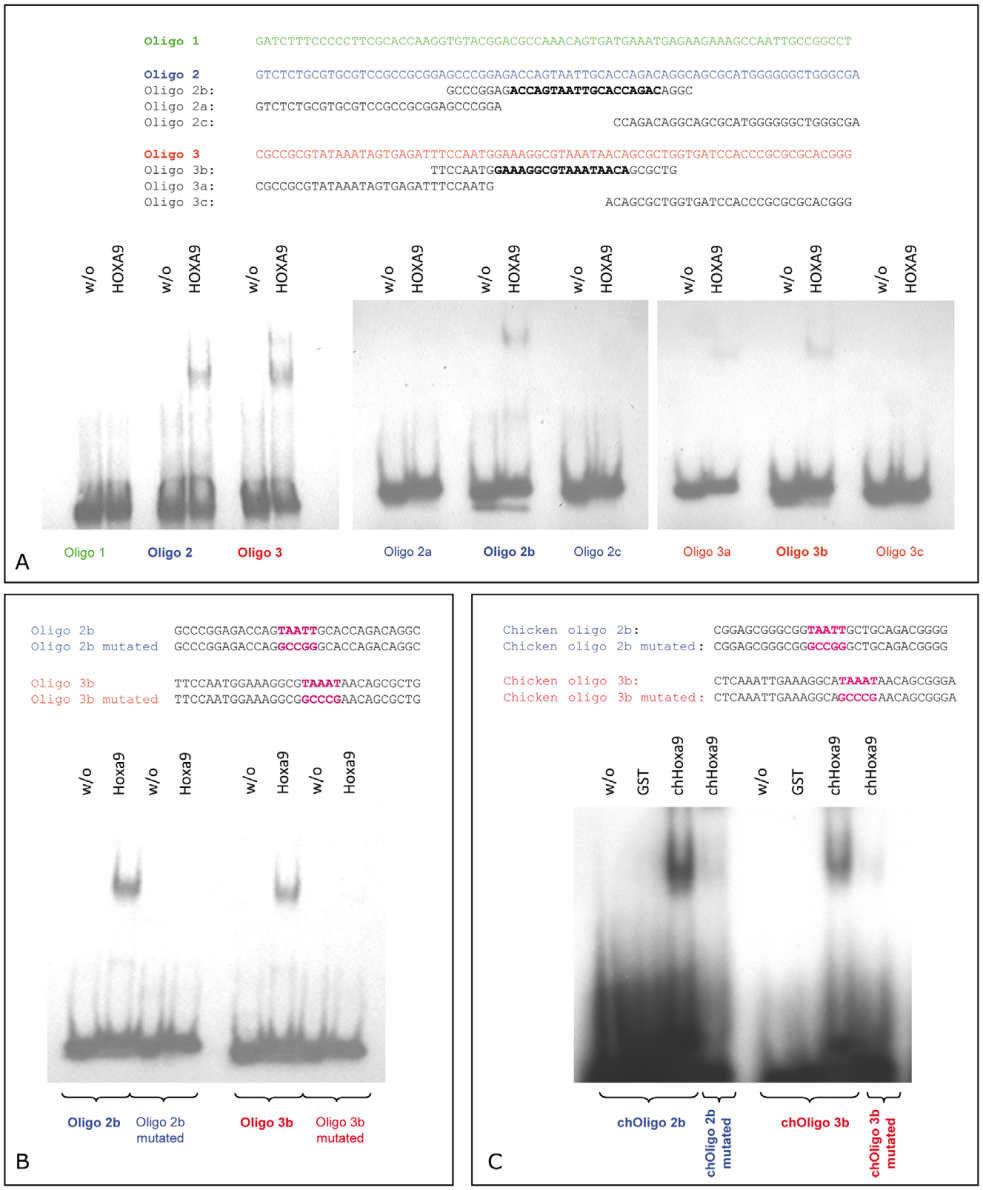
EMSA experiments to confine the exact binding sites of HOXA9 within the *SHOX* promoter 2. (A) Division of the *SHOX* promoter 2 sequence into three DNA oligos (green, blue, red) of similar lengths. Upon addition of purified GST-tagged HOXA9 protein, oligo 2 (blue) and oligo 3 (red) were able to bind HOXA9 (left panel). Further subdivision of oligo 2 and 3 into three overlapping oligos of 31 bp each revealed that only oligo 2b and 3b can bind to HOXA9, thus narrowing down the binding sites to two sequences of 31 bp each (middle and right panel). (B) Mutations of five nucleotides in oligo 2b or 3b, respectively, inhibited the binding of HOXA9. (C) EMSA experiments confirm the binding sites of cHoxa9 to the chicken *Shox* promoter. ChOligo 2b and 3b are homologous to the human oligos 2b and 3b that were used in the EMSA experiments in (A). Both chOligo 2b and 3b were able to bind cHoxa9 protein. Mutations of five nucleotides in chOligo 2b and 3b, respectively, largely inhibited the binding of cHoxa9. As a control, oligos were incubated without protein (w/o) or with GST alone, where no shift was observed.

**Figure 4:**
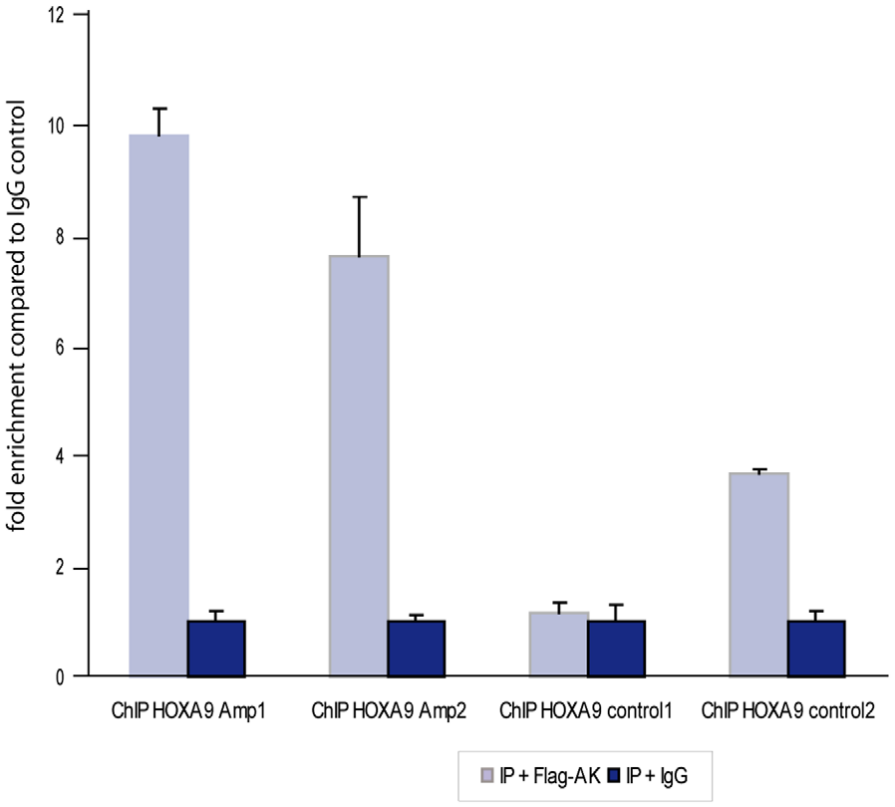
qRT-PCR of precipitated DNA of a chromatin immunoprecipitation (ChIP) experiment. ChIPs were performed from U2OS cells transfected with *HOXA9*-wt-Flag using an α-Flag-Antibody or mouse IgG as control, respectively. Samples of immunoprecipitated DNA were checked for an enrichment of the putative binding sites compared to randomly selected sequences residing in 0.8 to 2.5 kb distance. In total, four primer pairs were established, two of which reside within *SHOX* promoter 2 containing potential HOXA9 binding sites, and two of which reside outside that region. For better comparability, the amount of DNA that was amplified out of the control sample (IgG precipitation) was set to 1. The two PCR products amplifying the potential HOXA9 binding sites (ChIP HOXA9 Amp1 and Amp2) show a higher enrichment of immunoprecipitated DNA compared to the control regions (ChIP HOXA9 Contr 1 and 2). ChIP HOXA9 Contr 1 and 2 both are residing more than 2 kb from the promoter.

### Quantitative Real Time RT-PCR Analysis

Quantitative real time RT-PCR (qRT-PCR) was carried out using the Applied Biosystems 7500 Real-Time PCR System and Absolute SYBR Green ROX Mix (Abgene). Each sample was run in duplicates. Relative levels of mRNA expression were calculated according to the delta-delta Ct method by normalization to the expression of two different housekeeping genes (succinate dehydrogenase complex subunit A (*SDHA*) and peptidylprolyl isomerase A (*PPIA*). All primers used are listed in Table S1.

### Reporter Constructs and Luciferase Assays

*SHOX* enhancers or different regions of the two *SHOX* CpG Islands were cloned upstream of a firefly luciferase reporter gene (Promega) into the pGL3-Promoter or pGL3-Basic vector, respectively. Primers used for cloning are listed in Table S1. For luciferase reporter gene assays, U2OS cells were seeded into 24 well plates and transfected with 200 ng firefly reporter construct and 200 ng of pcDNA4/TO-*HOXA9* or pcDNA4/TO-*HOXD9* or the respective mutants. Luciferase activity was measured in triplicates 24 h after transfection using the Dual-Luciferase Assay Kit (Promega) according to the manufacturer’s protocol. This kit uses two different types of luciferase vectors, one of which serves as a control for normalization. In the case of *HOXA9* however, several luciferase control vectors reacted with an unspecific increase of luciferase activity upon the overexpression of *HOXA9* and were therefore not usable for normalization. Therefore, the presented data refer to the absolute activity of firefly luciferase. Experiments were repeated at least three times in triplicates with consistent results and representative data were shown.

**Figure 5:**
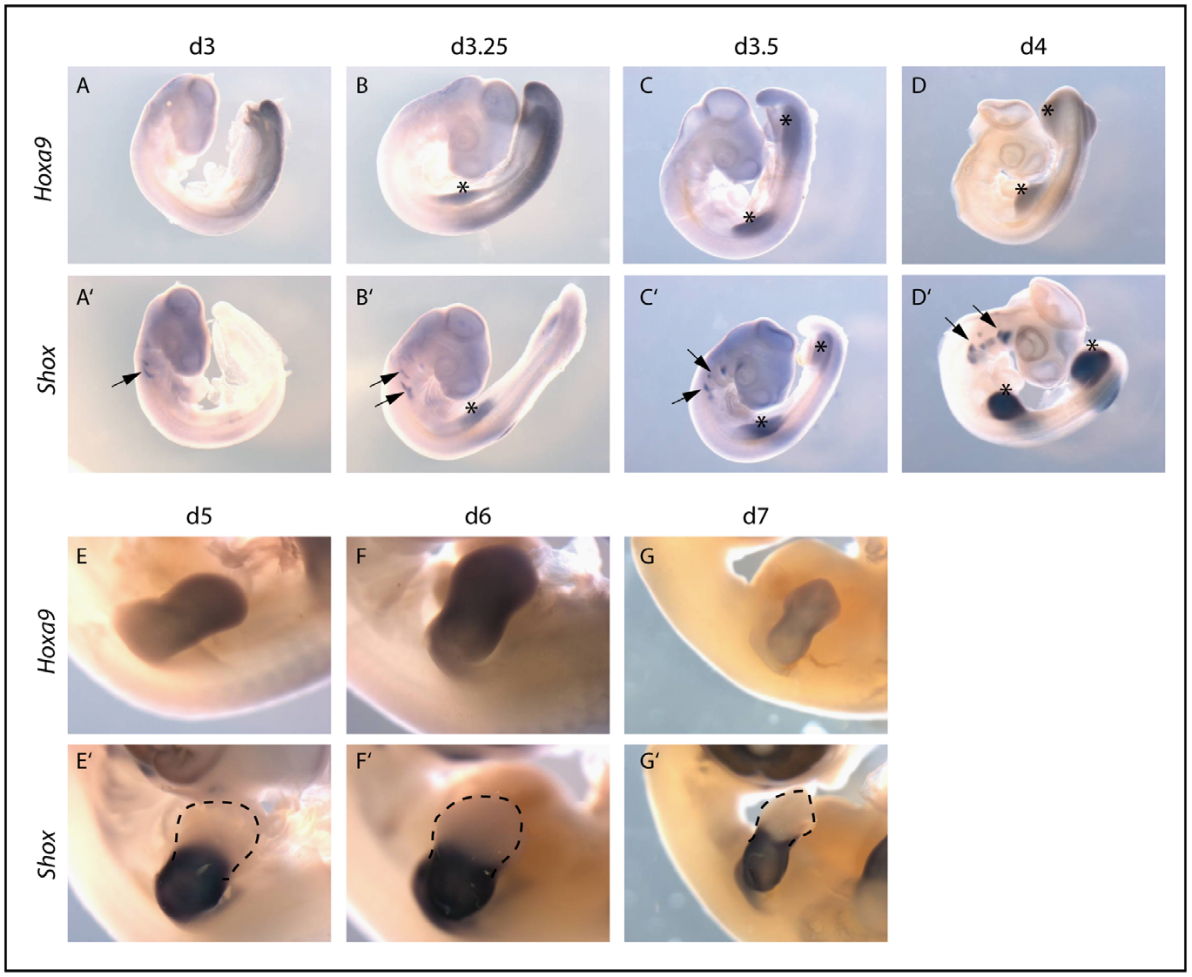
*In situ* hybridizations for *Hoxa9* and *Shox* in chicken embryos (d3-d7). The whole body is imaged for d3 to d4 embryos. Emerging limb buds are marked by an asterisk, pharyngeal arches are pointed by an arrow (A–D, A’-D’). For d5–d7 embryos, the right wing bud is presented to provide a detailed view of expression in the limb bud only (E–G, E’-G’). *Hoxa9* is expressed very early during embryonic development: expression is seen in d3 embryos along the vertebral axis of the posterior part of the body. In limb buds, expression starts at d3.25 (B) and persists until d6 (C–F). *Hoxa9* is expressed uniformly in the mesenchyme of the limb buds (A–G). *Shox* is also expressed during early embryonic stages and is already visible in the pharyngeal arches of d3 embryos (A’). With the outgrowth of the limb buds at d3.25 (B’), expression is also seen in wing and leg buds. Until stage d4, expression is seen in the whole limb bud (C’-D’); in later stages, expression is restricted to the middle segments of the limb buds (E’-G’). By stage d7, expression also begins to appear along the digital rays of the autopod (G’). Expression in the pharyngeal arches persists during all developmental stages analyzed (A’-G’).

### Electrophoretic Mobility Shift Assays

Electrophoretic mobility shift assays (EMSAs) were performed as previously reported [1]. For the binding reaction, ^32^P-labeled, double-stranded DNA oligonucleotides were used together with purified, bacterially expressed recombinant GST-HOXA9/GST-cHoxa9. Sequences of oligonucleotides are listed in Table S1. A total of 10 fmol of ^32^P-labeled probe was incubated with purified HOXA9 protein in a buffer containing 15 mM N-(2-hydroxyethyl)piperazine-N-(2-ethanesulfonicacid) (HEPES) pH 7.5, 60 mM NaCl, 1 mM ethylenediamine tetra acetate (EDTA), 0.5 mM dithiothreitol (DTT), 0.05% Nonidet P-40 (NP-40), 7.5% glycerol, 4 mM spermidine, 0.25 mg/ml bovine serum albumin (BSA) and 0.5 mg poly(dI/dC). Samples were loaded on native 5% polyacrylamide gels and electrophoresed in 0.25x TBE at 100 V for 50 min. Gels were dried and exposed for autoradiography.

### Chromatin Immunoprecipitation (ChIP)

A total of 1×10^7^ U2OS cells were transfected with pcDNA4/TO-HOXA9-FLAG. 24 to 48 h post-transfection, ChIP was carried out as described in [16] using anti-FLAG monoclonal antibody (Sigma, F1804).

### Chicken *in situ* Hybridization

Whole mount *in situ* hybridizations of chicken embryos were performed as described previously [17]. Riboprobes were generated and digoxigenin-labeled by *in vitro* transcription (DIG RNA Labeling Mix, Roche) of PCR products amplified out of chicken cDNA, using the primers listed in Table S1.

**Figure 6:**
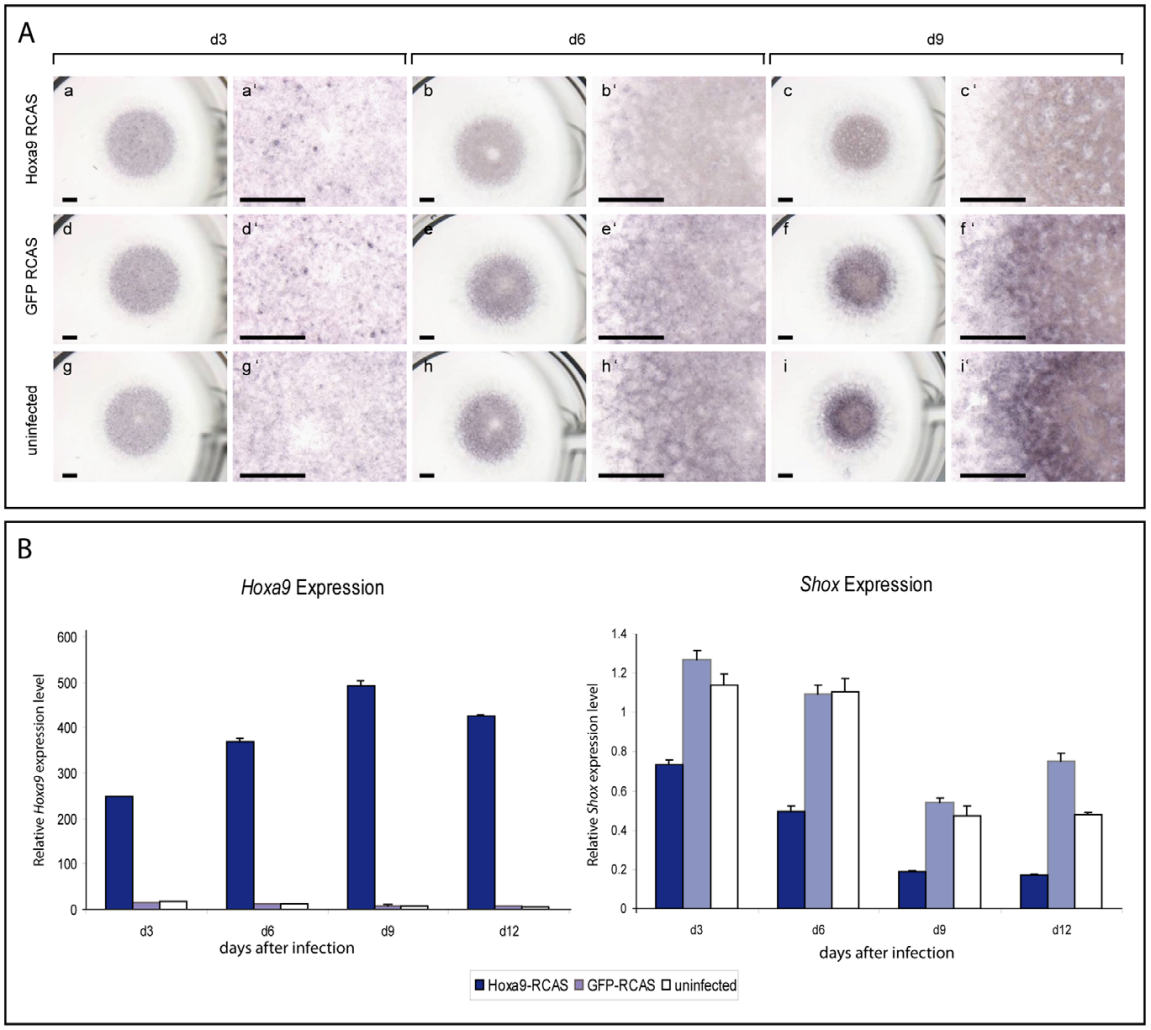
Analysis of the effect of Hoxa9 overexpression in chMM cultures by qRT-PCR and *in situ* hybridization. (A) *In situ* hybridization on chMM cultures (d3–d9). (a–i) overview of cultures; (a’-i’) detailed view. Especially in d6 and d9 cultures, *Hoxa9* infected cultures (a–c’) exhibit a generally weaker *Shox* expression compared to the control cultures (d–f’ and g–I’). Scale bar = 1000 µM. (B) Left panel: qRT-PCR analysis of *Hoxa9* expression levels after virus-induced Hoxa9 overexpression. Infection with Hoxa9-RCAS leads to a strong increase of *Hoxa9* expression for all time points analyzed. Right panel: qRT-PCR analysis of *Shox* expression levels in the corresponding samples. For all time points analyzed, *Shox* expression is reduced in the cultures that have been infected with Hoxa9 virus.

### Chicken Micromass Culture (chMM)

Limb buds of 30 chicken embryos (HH stage 24) were removed, pooled and washed twice with Hanks Balanced Salt Solution (HBSS; Gibco). To get a single cell suspension, the limb buds were digested with dispase (Gibco, 3 mg/ml in HBSS) for 15 min with continuous shaking at 37°C, washed multiple times to remove ectodermal tissue and incubated with digestion solution (0.1% [w/v] Collagenase type Ia [Sigma], 0.1% [w/v] Trypsin [Gibco], 5% FBS [PAA] in DPBS [Gibco]) at 37°C for 30 min. After addition of chMM medium (DMEM/HAM’S F12 [Gibco] with 10% FBS [PAA], 0.2% Chicken Serum [Sigma], 1% L-Glutamin [Gibco] and Penicillin/Streptomycin [Gibco]), the resulting suspension was passed through a 40 µm nylon filter (BD) to remove cell aggregates. Cells were counted, and the suspension was adjusted with chMM medium to a concentration of 2×10^7^ cells/ml. Aliquots of this suspension were treated with 50 µl of RCAS(BP) (replication-competent ASLV [avian sarcoma-leukosis virus] long terminal repeat [LTR] with a splice acceptor and Bryan polymerase) virus solution (5×10^7^ virus particles/ml) RCAS(BP)-Shox, RCAS(BP)-Hoxa9, RCAS(BP)-GFP or 50 µl of virus-free medium. 10 µl drops were seeded into 24 well plates and incubated for 2 h at 37°C, 5% CO_2_ and 95% humidity. Subsequently, 2 ml of chMM medium were added and cells were cultivated, forming round cultures which were harvested after 3, 6, 9 and 12 days with medium change every two days. For qRT-PCR, RNA was isolated and processed as described above. For *in situ* hybridizations, cultures were fixed with 4% paraformaldehyde for 30–60 min and subjected to whole mount *in situ* hybridization as described above with probes for *Shox* or *Hoxa9*. For Alcian blue staining, chMM cultures were washed with PBS and fixed in Kahles fixing solution (30% EtOH, 0.4% PFA, 4% glacial acetic acid) at RT for 15 min. After washing with PBS, cultures were incubated in 0.05% Alcian blue solution (0.05 Alcian blue in 0.1 M HCl) at RT for 24 h and then rinsed with distilled water.

## Results

### *In silico* Analysis of *SHOX* CpG Islands and Enhancer Sequences for Transcription Factor Binding Sites

To identify potential transcription factors that can bind to *SHOX* regulatory elements, we carried out *in silico* transcription factor binding site predictions using MatInspector (Genomatix Software GmbH) [18], [19]. The analysis of regulatory sequences comprised the *SHOX* upstream enhancers CNE-5, CNE-3 and CNE-2 [12], the three downstream enhancers CNE+4, CNE+5 and CNE+9 [13] and two CpG islands in the 5′ region of *SHO*X which contain the two identified *SHOX* promoters (CpG 1 chrX/Y:504,564-505,326; CpG 2 chrX/Y:510,430–512,197; as annotated by the UCSC browser; NCBI36/hg18) [10]. For the binding site analyses, we only considered sequences within the CpG islands or the enhancers that were 100% conserved (identical) among human, dog, opossum, chicken, frog and zebrafish. Analysis of these conserved sequences of about 20–30 bp yielded predictions for 10–20 binding partners each. For five out of six analyzed enhancer sequences (CNE-3, CNE-2, CNE+4, CNE+5 and CNE+9) and for the CpG island 2, homeobox transcription factor binding sites were predicted. These predictions included binding site predictions for HOX proteins in general and in particular HOXA9, together with the typical HOX cofactors PBX1 and MEIS1. This accumulation of predicted HOXA9 binding sites together with the fact that certain *HOX* genes (similar to *SHOX*) are well-known to play an essential role during limb development (e.g. [20], [21], [22], [23], [24]), rendered HOXA9, PBX1 and MEIS1 as the most interesting binding candidates.

*HOX* genes encode for a highly conserved family of closely related transcription factors playing an essential role during the formation of the main body axes [25] and the axes of appendicular structures such as limbs and genital buds [26], [27]. During limb development, especially the 5′ genes of the *HOXA* and *HOXD* cluster (*HOXA9-HOXA13* and *HOXD9-HOXD13*) are of special importance for the patterning of the proximo-distal axis of the developing limbs and are expressed in well-defined segmental domains along the limbs (reviewed in [20]). Here, the expression pattern of *Hoxa9* overlaps with the *Shox* expression [9], [21], [28] (Figure S1), which prompted us to analyze HOXA9 and the HOX cofactors PBX1 and MEIS1 in functional studies.

### Overexpression Studies Identify HOXA9 as a Potential Regulator of *SHOX* Expression

*HOXA9* and the genes encoding for the HOX cofactors *PBX1* and *MEIS1* were PCR-amplified out of human U2OS cell line cDNA, Flag-tagged C-terminally and cloned into the eukaryotic expression vector pcDNA4/TO (Invitrogen).

*HOXA9* or the cofactors, respectively, were then individually transfected into U2OS cells and expression was verified on RNA (qRT-PCR) as well as protein level (Western Blot using an α-Flag antibody; data not shown). The influence of the overexpression of these transcription factors on *SHOX* expression was then analyzed by qRT-PCR. The transcription factor *SHOX* is generally only expressed at very low levels in U2OS cells (like in all other cell lines and cultured primary cells) [29]. Overexpression of *MEIS1* and *PBX1* did not change the *SHOX* expression level compared to that of the control transfection with the empty overexpression vector. In contrast, a consistent increase of *SHOX* expression was detected after *HOXA9* overexpression for all time points analyzed (12 h, 24 h and 48 h after transfection).

We therefore focused on the analysis of the regulatory potential of HOXA9 on *SHOX* expression. To rule out unspecific protein mass effects, which might have elicited the observed increase of *SHOX* expression after *HOXA9* overexpression, we generated two different *HOXA9* mutants by introducing point mutations into the most conserved parts of the homeodomain (HOXA9 mut1: K223E; HOXA9 mut2: K223E, N256del, R257P, R258G). In subsequent time course experiments, only HOXA9 wild type but not the two mutants were shown to increase *SHOX* expression, arguing for a HOXA9-dependent upregulation of *SHOX* expression (Figure 1).

### HOXA9 Binds to Distinct Sites within *SHOX* Promoter 2 and Activates *SHOX* Expression

To determine which of the *SHOX* regulatory sequences are targeted by HOXA9, we performed luciferase reporter gene assays. For that purpose, we cloned the known six limb specific *SHOX* enhancer elements (CNE-5, CNE-3, CNE-2, CNE+4, CNE+5 and CNE+9) [12], [13] into the pGL3-Promoter vector and the two *SHOX* CpG islands into the vector pGL3-Basic. An overview of the genomic location of the *SHOX* regulatory elements is given in Figure 2A. The constructs were then cotransfected in U2OS cells together with *HOXA9* wild type or *HOXA9* mutant expression constructs or the empty expression vector, respectively. The results in Figure 2B indicate that no specific increase of firefly luciferase activity was detected in cells cotransfected with the enhancer constructs. However, the analysis of the CpG islands revealed a specific and very strong increase of firefly luciferase activity for CpG2 and a specific but mild increase for CpG1 (Figure 2C, left and middle panel), suggesting a regulatory role of CpG2 in the HOXA9-mediated activation of *SHOX* expression.

As a control, we also investigated whether the paralog HOXD9 can also affect *SHOX* expression and carried out luciferase reporter assays using HOXD9 and two HOXD9 mutants (HOXD9 Mut1: K292E; HOXD9 Mut2: K292E, R326P, R327G). As shown in Figure 2C (right panel), HOXD9 was not able to increase luciferase activity comparable to the effect caused by *HOXA9* overexpression, arguing for a specific effect of HOXA9 on *SHOX*.

Due to the strong upregulation of luciferase activity for CpG2 upon *HOXA9* overexpression, we focused on CpG2. Transfection of serial deletion constructs of CpG2 enabled us to narrow down the strongest HOXA9 responsive element of CpG2 to an interval of 465 bp (CpG2 part 2a) containing the known *SHOX* promoter 2 (298 bp) (Figure 2 D, E). We then used three DNA sequences of equal lengths (75–77 bp) within *SHOX* promoter 2 and performed electrophoretic mobility shift assays (EMSA). Two of these oligonucleotides (oligo 2 and oligo 3) showed a retarded gel migration after incubation with purified GST-tagged HOXA9, indicating a binding of HOXA9 protein to these sequences (Figure 3 A, left panel). A further subdivision into three partially overlapping oligonucleotides of the same lengths (oligos 2a to 2c and oligos 3a to 3c) revealed that the oligos 2b and 3b (each 31 bp) were sufficient for GST-HOXA9 binding (Figure 3 A, middle and right panel). Oligo 2b and 3b both comprise AT-rich palindrome sequences which represent classical HOX binding sites [30]. Mutation of these AT motifs prevented binding of HOXA9 to the oligos (Figure 3 B), thus confirming that these palindromic sequences are essential for HOXA9 binding in this region. The specific binding of HOXA9 to *SHOX* promoter 2 in U2OS cells was also validated by chromatin immunoprecipitation (ChIP) where the sequences identified by luciferase assays and EMSA (*SHOX* promoter 2) were enriched in HOXA9-immunoprecipitated DNA (Figure 4).

Usage of *SHOX* promoter 2 has been previously demonstrated to lead to a high translational activity of *SHOX* mRNA lacking the non-coding exon 1, whereas usage of *SHOX* promoter 1 gives rise to an mRNA with a long 5′UTR containing exon 1 [10]. We therefore carried out qRT-PCR experiments on HOXA9-transfected U2OS cells to discriminate between *SHOX* mRNA isoforms that have been transcribed from promoter 1 or 2, respectively, using a primer pair spanning from exon 1 to exon 2. No increase of the *SHOX* exon 1 containing isoform was found using exon 1 specific primers (Figure S2), validating that HOXA9-mediated activation of *SHOX* transcription is accomplished via promoter 2.

Taken together, these results show that HOXA9 can activate *SHOX* expression in U2OS cells by binding to distinct sites within *SHOX* promoter 2.

### Analyses in Chicken Micromass Cultures Reveal a Negative Regulation of *Shox* by Hoxa9 during Cartilage Differentiation

To further evaluate the regulation of *SHOX* by HOXA9 *in vivo*, we used chicken embryos as a model since *SHOX* has no ortholog in mouse or other rodents. We first compared the expression patterns of *Hoxa9* and *Shox* in the developing embryo, especially focusing on the expression in developing limb buds.

Whole mount *in situ* hybridization showed that *Hoxa9* expression is already present in day3 (d3) embryos along the vertebral axis of the posterior part of the body (Figure 5). In limb buds, *Hoxa9* expression starts at d3.25, is uniformly expressed in the limb bud mesenchyme and weakens after d6. *Shox* expression is first seen in the pharyngeal arches and with the outgrowth of the limb buds at d3.25, expression is also detected in wing and leg buds. Until d4, expression broadens to the whole limb bud and then gets confined to the middle limb segments during later stages. By d7, *Shox* expression is also found along the digital rays of the autopod (Figure 5). Hence, *Hoxa9* and *Shox* are coexpressed in the limb buds, which is a requirement for a regulatory relationship of the two genes.

To study the HOXA9/SHOX regulatory mechanisms during cartilage formation, we used chicken micromass cultures (chMM) as previously described [16]. ChMM are an *in vitro* culture system that simulates the processes occurring during endochondral ossification and limb development. In this system, cultures of mesenchymal cells that were isolated from embryonic chicken limbs at HH-stage 24 spontaneously differentiate into chondrocytes and connective tissue [31], [32]. The cultures can be infected with RCAS, a replication-competent retroviral vector system, enabling overexpression of a gene of interest in chicken tissue. In our study, chMM were infected with RCAS *Hoxa9*, RCAS *Shox,* RCAS *GFP* or were left uninfected.

To analyze the effect of *Hoxa9* overexpression on *Shox,* we performed whole mount *in situ* hybridization on d3, d6 and d9 cultures. A strong *Hoxa9* expression was observed in all *Hoxa9*-infected cultures, indicating good transduction efficiencies (data not shown). After 6 and 9 days of differentiation, *Shox* expression was reduced in *Hoxa9*-infected cultures compared to control cultures (Figure 6 A) pointing to a negative regulation of *Shox* by Hoxa9. To quantify this effect, RNA from d3, d6, d9 and d12 cultures was isolated and gene expression levels were determined by qRT-PCR. For all time points analyzed, a 2 to 3-fold lower *Shox* expression in the *Hoxa9*-infected cultures compared to control cultures was detected (Figure 6 B). In addition, morphological analyses of the differentiation status of the chMM cultures were carried out to further evaluate the observed negative regulation of *Shox* by Hoxa9. After 3 days of cultivation, all cultures had formed aggregates of undifferentiated chondrocytes. Morphological differences between the control cultures and the *Hoxa9*- or *Shox*-infected cultures became apparent after 6 days of cultivation. While *Shox*-infected cultures grew more compactly and higher, Hoxa9-infected cultures were flattened as compared to control cultures (Figure S3). These opposing effects of Hoxa9 and Shox on ChMM differentiation again argue for a negative regulation.

In a last step, we wanted to know if the regulation in human and chicken is accomplished by the same binding sites. Interestingly, the two identified HOXA9 binding sites within human *SHOX* promoter 2 reside in a highly conserved region. Compared to chicken, the identified target sequences (oligo 2b and 3b) exhibit a conservation of 74% and 77%, respectively, with the palindromic AT-rich region being conserved to 100%. We therefore decided to carry out additional EMSA experiments with purified chicken Hoxa9 protein and the homologous chicken *Shox* promoter 2 target sequences (chOligo 2b and 3b). Here, we could show that cHoxa9 is able to bind to the homologous DNA sequences within the promoter whereas mutated chicken oligos largely prevented the binding (Figure 3C). Thus, it is very likely that the regulation of *Shox* by Hoxa9 in chMM cultures is accomplished by the same binding sites as in human U2OS cells.

In summary, *in situ* hybridization, qRT-PCR and differentiation studies of *Hoxa9*- and *Shox-* infected cultures indicate a negative regulation of *Shox* by Hoxa9 in chicken micromass cultures. EMSA experiments have shown that the same binding sites within the human or chicken promoter can be used.

## Discussion

The homeobox gene *SHOX* is known to play a key role during limb development, and mutations or deletions lead to the limb malformations seen in LWD and Langer syndrome or to short stature without limb anomalies in patients with idiopathic short stature [3], [4], [5], [33]. Limb malformations can be due to defects in the coding region of the gene but also to deletions of regulatory elements on either side of the *SHOX* gene [11], [12], [13], [34], [35]. These findings demonstrate that the correct function of SHOX does not only depend on the correct protein sequence and folding but also on accurate regulatory processes that direct the appropriate expression of *SHOX*. The aim of this study was to identify regulators of *SHOX* expression that interact with *SHOX* enhancer or promoter elements. These studies remain challenging as most of the standard techniques for the analysis of DNA-protein interactions such as ChIP-Seq (chromatin immunoprecipitation) or SELEX (**S**ystematic **E**volution of **L**igands by **EX**ponential Enrichment) are directed to identify DNA sequences where the protein of interest can bind to. However, for the identification of proteins that bind to a given DNA sequence, as in our case, no standard high throughput methods have been developed. We therefore initially used an *in silico* approach and identified HOX proteins as putative binding partners of *SHOX* regulatory elements. Subsequent overexpression experiments then revealed that HOXA9 activates *SHOX* expression by binding to promoter 2 in U2OS cells (Due to the very low expression level of *SHOX* in cell lines and tissues, a resulting down-regulation of *SHOX* after HOXA9 depletion is impossible to detect with confidence. This low expression level is a characteristic for some transcription factors, including SHOX and is a limiting factor for certain experimental approaches, including knock-down studies). Also in chicken micromass cultures, *Shox* expression was regulated by Hoxa9. In contrast to the findings in U2OS cells, Hoxa9 acted as a repressor of *Shox* expression in chMM, suggesting that, dependent on the cellular environment, HOXA9 can either function as an activator or a repressor of *SHOX*.

All 39 human HOX proteins share a highly conserved homeodomain which recognizes the same core DNA consensus sequence [30]. The individual binding specificity of each HOX protein is accomplished through cooperative binding with cofactors, whose abundance is dependent on the cellular environment and subject to spatio-temporal regulation. Depending on the activation of different cofactors and signaling pathways in different tissues, individual *HOX* genes can switch from activators to repressors of gene transcription [30], [36]. The most common HOX cofactors are the TALE-homeodomain proteins PBX1 and MEIS1, but further cofactors have been identified recently and are expected to be identified in the future [37]. We also have examined the influence of cofactors on HOXA9-mediated *SHOX* regulation in U2OS cells by coexpressing *HOXA9* with *PBX1* and/or *MEIS1* but no differences in the *SHOX* expression level were detected compared to when solely *HOXA9* was expressed (data not shown). Thus, we speculate that other, additional cofactors may modulate the effect of Hoxa9 in U2OS and chMM cultures. We also cannot exclude that other HOX proteins might influence *SHOX* expression levels in cells or model systems other than the ones assessed in our study.

By luciferase assays, we have shown that HOXA9 has an activating effect on *SHOX* promoter 2 in U2OS, which was not seen in control experiments where HOXA9 mutants or HOXD9 were used, confirming the specificity of the binding. In EMSA experiments, the HOXA9 binding site could be narrowed down to two AT-rich palindrome sequences of 31 bp within promoter 2. These sequences were specifically bound by HOXA9 but not by other homeodomain proteins such as SHOX. The identification of two HOXA9 binding sites within *SHOX* promoter 2 is concordant with recent findings where homotypic clusters of transcription factor binding sites for the same transcription factor within promoter regions were identified [38]. This clustering of transcription factor binding sites is especially prevalent in the proximal promoter regions of transcription factor genes with nearly two thirds of these genes exhibiting multiple binding sites for other transcription factors.

A similar clustering of binding sites is also seen in the chicken *Shox* promoter where the two binding sites identified in human are highly conserved. In total, the identified target sequences (oligo 2b and 3b) exhibit a conservation of 74% and 77%, respectively, with the palindromic AT-rich region being conserved to 100%. As we could show a binding of cHoxa9 protein to the homologous DNA sequences within the chicken *Shox* promoter, it is very likely that the regulation of *Shox* by Hoxa9 in chMM cultures is accomplished by the same binding sites as in human U2OS cells.

Formation of limb buds and their continued outgrowth depend on an integrated highly conserved network of multiple different signaling molecules and gene regulators. The two major signaling centers during limb development are the Apical Ectodermal Ridge (AER) and the Zone of Polarizing Activity (ZPA), which mediate their activities by specific secreted signaling molecules in the limb buds. Fgfs (**F**ibroblast **g**rowth **f**actors) are the main signaling factors produced by the AER [39], [40], whereas the ZPA mainly acts via the secreted morphogen Shh [41]. Experiments in chicken have shown that *Shox* expression is regulated by different Fgf molecules but independent from Shh signaling [6], [9]. Thus, *Shox* expression depends on AER but not on ZPA signaling.

Correct limb bud formation also requires specific expression of *Hox* genes of the A and D clusters, in particular those of the 5′ end (i.e., *Hoxa9* to *Hoxa13* and *Hoxd9* to *Hoxd13)*. Studies in mice showed that loss-of-function mutations in these genes strongly impair limb morphology with patterning defects generally affecting particular regions of the developing limb (reviewed by [20]). Hox genes are therefore thought to determine the individual segment identity of the limb skeleton. *Hoxa9^−/−/^Hoxd9^−/−^* double knockout mice display a phenotype in the stylopod, which is shortened and malformed compared to wild type mice [21]. However, due to the lack of a *SHOX* ortholog in the mouse genome, these results cannot be directly transferred to the human system. In humans, only a few *HOX* genes have been associated with a disease phenotype. While it is known that mutations in HOXA13 or HOXD13 can cause specific limb phenotypes, such as hand-foot-genital-syndrome [42], [43] or synpolydactyly [44], [45], no *HOXA9*-related disease phenotype has been identified in patients so far, leaving the role of HOXA9 during human limb development unclear. *SHOX* mutations in humans mainly affect the bones of the zeugopod region (LWD, Langer syndrome) which is in conformity with the most prominent expression of the chicken and human *SHOX* gene in the middle part of the developing limb [6], [9]. Due to the overlap of expression of *Shox* and *Hoxa9* in the developing chicken limb it is conceivable that HOXA9 and SHOX are in a regulatory relationship during human limb development. The negative regulation of *Shox* by Hoxa9 observed in chMM (a model for chondrogenesis and bone formation) argues for a likewise negative regulation of these processes during limb development; however the expression data in chicken suggest that the regulation is not exhaustive, at least not at the surface of the limb.

The negative regulation of *Shox* by Hoxa9 observed in chMM is also supported by the model of Zakany and Duboule [20] where *Hox* genes are proposed to play fundamental roles in determining AER and ZPA functions during mammalian forelimb development. According to this hypothesis, *Hox* genes of group 10–13 are involved in the induction of the ZPA, whereas the lower *Hox* genes are important for AER formation where in particular *Hoxa9* is known to be able to induce *Fgf* expression. Since it has been shown that *Shox* expression is downregulated by AER signaling molecules (Fgfs and Bmps), *Shox* might be negatively regulated by Hoxa9 in both a direct (by binding to the *Shox* promoter) and indirect manner (by inducing AER signaling).

In summary, we have successfully identified a regulator of SHOX expression and provide evidence for a direct regulation of SHOX by HOXA9. These findings may further contribute to unravel the diversified regulatory networks during limb development and may also help to improve our knowledge of the etiopathogenesis of short stature.

## Web addresses

UCSC genome browser: http://genome.ucsc.edu/

Genomatix software suite with MatInspector program: http://www.genomatix.de/en/produkte/genomatix-software-suite.html

## Supporting Information

Figure S1 **Expression pattern of Shox and the** ***Hox*** **genes important during limb development in a d11.5 mouse embryo.** (A, taken from Fromental-Ramain et al. 1996). (A) Schematic spatial expression of 5′Hox genes in the developing mouse limb at stage E11.5 dpc (B) Expression pattern of *Shox* in a chicken embryo of the corresponding developmental stage (d5). An overlap in the expression domain of Shox is seen especially for Hoxa9, Hoxa10, Hoxd9 and Hoxd10 when comparing the expression patterns of *Hox* genes in mouse and *Shox* in chicken.
(TIF)

Figure S2 **RT-PCR using primer spanning SHOX exon 1 to 2 confirms the regulation via promoter P2.** (A) Schematic representation of the two isoforms that are transcribed from promoter P1 or P2, respectively. With qPCR experiments using a primer pair spanning from exon 1 to 2 (depicted by the two opposing arrows) it is possible to discriminate between the two isoforms. (B) RT-PCR using primers spanning exon 1 to 2 using the same cDNA samples in which we had seen an increase of *SHOX* expression after *HOXA9* overexpression (for comparison see Figure 1; here, a primer pair spanning exon 5 to 6 was used). No increase of *SHOX* expression can be seen for exon 1 to 2 confirming that HOXA9 mediated activation of *SHOX* transcription is accomplished via promoter 2.
(TIF)

Figure S3 **Alcian Blue staining of chMM cultures.** Uninfected cultures (A–D) and GFP-RCAS infected (E–H) control cultures show a similar morphology during differentiation, but differ from the morphologies of Shox-RCAS (I–L) and Hoxa9-RCAS (M–P) infected cultures. After 3 days of cultivation, all cultures have formed aggregates of undifferentiated chondrocytes (A, E, I, M). After 6 days of cultivation, morphological differences between the control cultures and the *Hoxa9*- or *Shox*-infected cultures become apparent indicating an opposing differentiation behavior. *Shox*-infected cultures grow more compactly and higher, Hoxa9-infected cultures grow flatter as compared to control cultures.
(TIF)

Table S1 **List of primers and oligonucleotides.** Listed are all primers and oligonucleotides that were used for cloning, mutagenesis, quantitative real time RT-PCR analysis, chromatin immunoprecipitation and in EMSA experiments.
(DOC)
