# Supplementary figures and images for: The Homeobox Transcription Factor HOXA9 Is a Regulator of SHOX in U2OS Cells and Chicken Micromass Cultures
Source: PLoS One. 2012 Sep 20;7(9):e45369. doi: 10.1371/journal.pone.0045369 (PMC3447975; doi:10.1371/journal.pone.0045369)

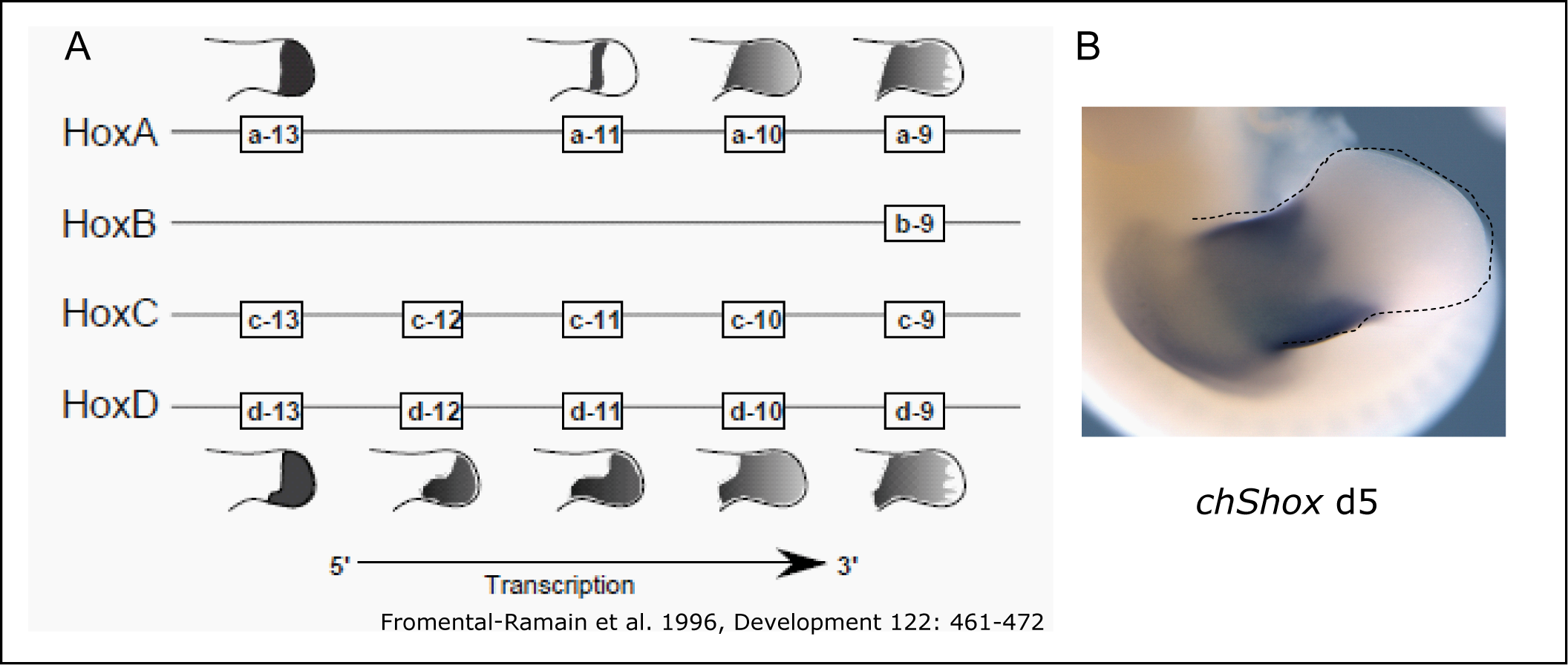

Supplement: Figure S1 — Expression pattern of Shox and the Hox genes important during limb development in a d11.5 mouse embryo. (A, taken from Fromental-Ramain et al. 1996). (A) Schematic spatial expression of 5′Hox genes in the developing mouse limb at stage E11.5 dpc (B) Expression pattern of Shox in a chicken embryo of the corresponding developmental stage (d5). An overlap in the expression domain of Shox is seen especially for Hoxa9, Hoxa10, Hoxd9 and Hoxd10 when comparing the expression patterns of Hox genes in mouse and Shox in chicken. (TIF) [file pone.0045369.s001.tif]

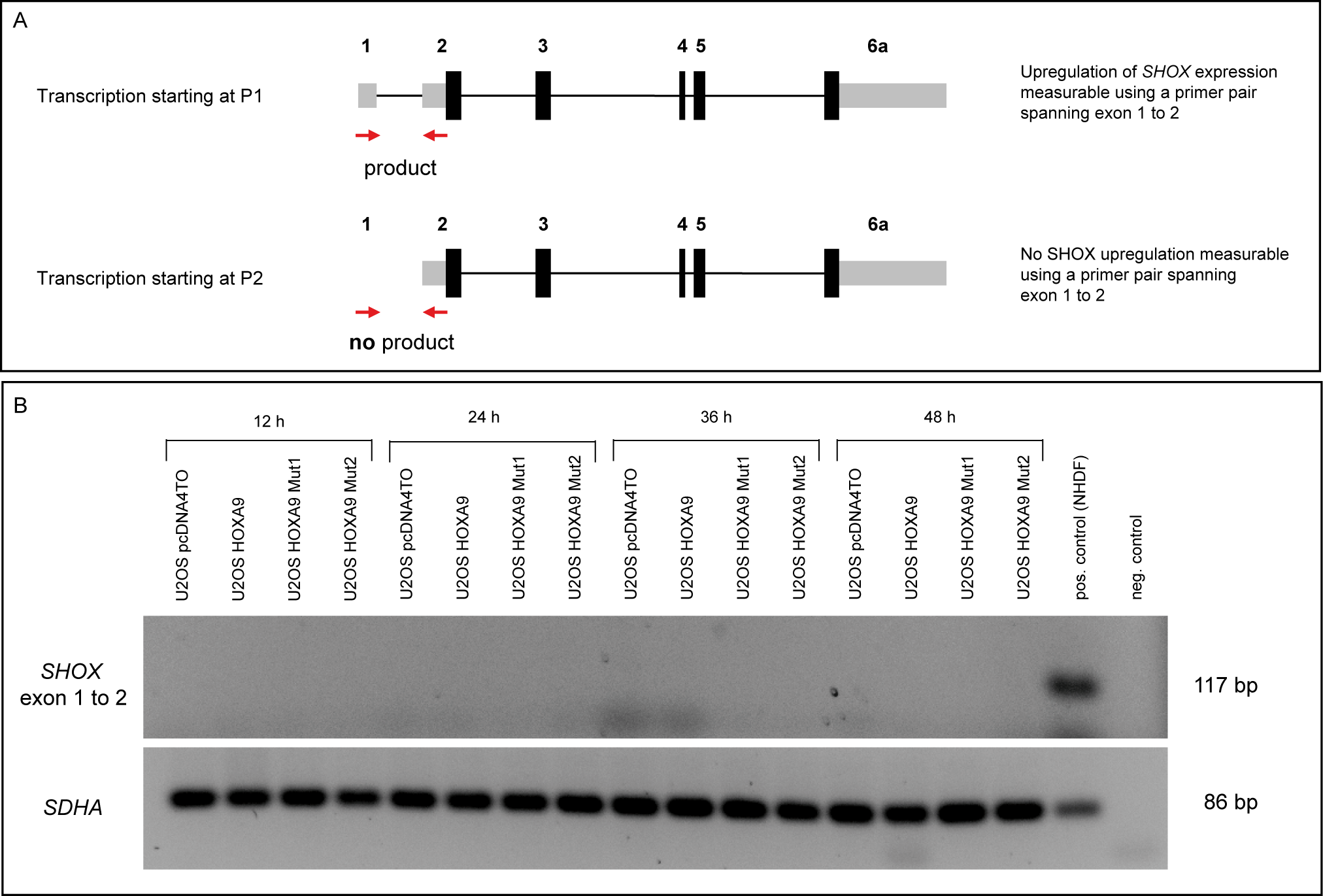

Supplement: Figure S2 — RT-PCR using primer spanning SHOX exon 1 to 2 confirms the regulation via promoter P2. (A) Schematic representation of the two isoforms that are transcribed from promoter P1 or P2, respectively. With qPCR experiments using a primer pair spanning from exon 1 to 2 (depicted by the two opposing arrows) it is possible to discriminate between the two isoforms. (B) RT-PCR using primers spanning exon 1 to 2 using the same cDNA samples in which we had seen an increase of SHOX expression after HOXA9 overexpression (for comparison see Figure 1; here, a primer pair spanning exon 5 to 6 was used). No increase of SHOX expression can be seen for exon 1 to 2 confirming that HOXA9 mediated activation of SHOX transcription is accomplished via promoter 2. (TIF) [file pone.0045369.s002.tif]

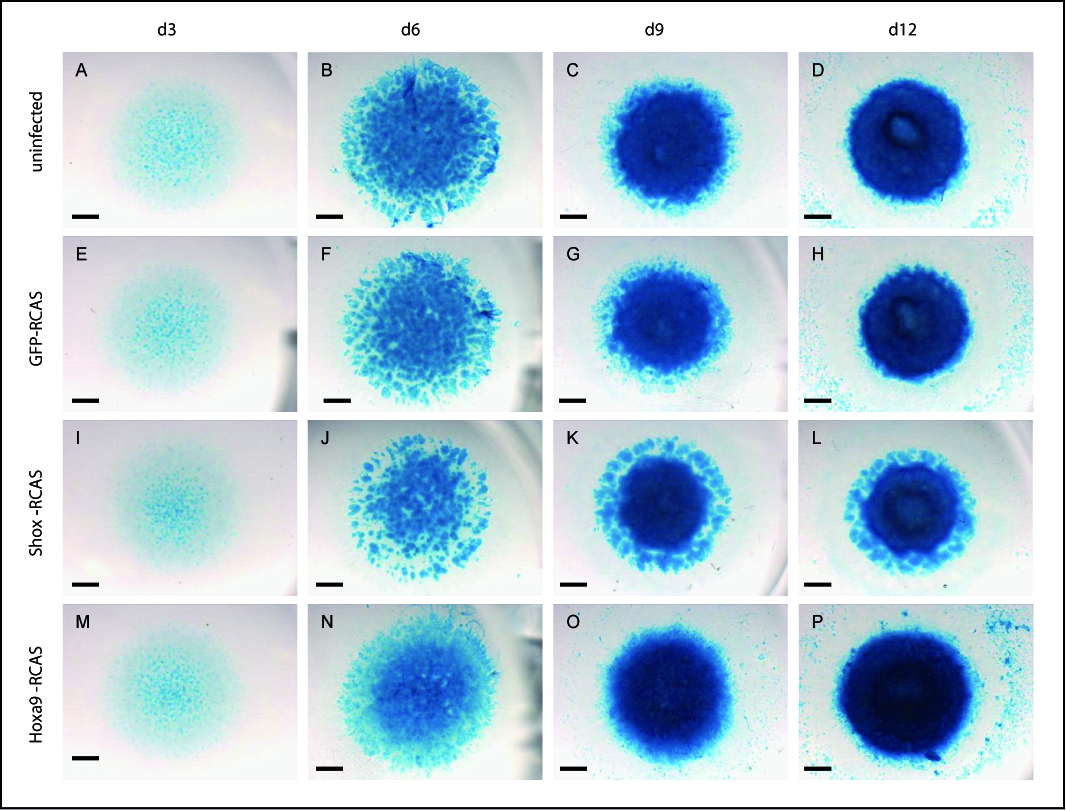

Supplement: Figure S3 — Alcian Blue staining of chMM cultures. Uninfected cultures (A–D) and GFP-RCAS infected (E–H) control cultures show a similar morphology during differentiation, but differ from the morphologies of Shox-RCAS (I–L) and Hoxa9-RCAS (M–P) infected cultures. After 3 days of cultivation, all cultures have formed aggregates of undifferentiated chondrocytes (A, E, I, M). After 6 days of cultivation, morphological differences between the control cultures and the Hoxa9- or Shox-infected cultures become apparent indicating an opposing differentiation behavior. Shox-infected cultures grow more compactly and higher, Hoxa9-infected cultures grow flatter as compared to control cultures. (TIF) [file pone.0045369.s003.tif]
