# Supplementary material for: The Homeobox Transcription Factor HOXA9 Is a Regulator of SHOX in U2OS Cells and Chicken Micromass Cultures
Source: PLoS One. 2012 Sep 20;7(9):e45369. doi: 10.1371/journal.pone.0045369 (PMC3447975; doi:10.1371/journal.pone.0045369)
Supplement: Table S1 — List of primers and oligonucleotides. Listed are all primers and oligonucleotides that were used for cloning, mutagenesis, quantitative real time RT-PCR analysis, chromatin immunoprecipitation and in EMSA experiments. (DOC) [file pone.0045369.s004.doc]

| **Primers for cloning of the Luciferase constructs** | |
| --- | --- |
| **Name** | **Sequence (5'® 3')** |
| CNE-3 for | GCCAGATCTCGAGGTGGATCAAAGTGTCA |
| CNE-3 rev | GGCGAATTCTGCTCTGCCATATCCTCAATC |
| CNE-4 for | GCGAGATCTTAGATAAGGGACCTCCTCTG |
| CNE-4 rev | CGCGGAATTCGATTTTCTGGCTGGAAATGG |
| CNE-2_MluI_For | CAGACGCGTACATGACAGCCGGGCCTCTG |
| CNE-2_XhoI_Rev | CAGCTCGAGGCGAGCCATAAAACAAGCTG |
| CNE-5_MluI_F | CGTACGCGTCAAACACGGAACAGCACACT |
| CNE-5_XhoI_R | GGTCTCGAGTCTCCGCCTCTTCGGCAGA |
| CNE+4_MluI_F | CACACGCGTTTTGCAGTGTTATGCACTCG |
| CNE+4_XhoI_R | CACCTCGAGCTGGTGTTCGGTCTCAGCTC |
| CNE+5_MluI_F | GACACGCGTCGTAGAATGCCAGAGATGAGG |
| CNE+5_XhoI_R | CAGCTCGAGTGGCTGTCGATGAACTTTGT |
| CNE+9_MluI_F | TGTACGCGTTATACTTTACTTCTTTGCTG |
| CNE+9_XhoI_R | TGTCTCGAGTTGTGTCTGCAGTGTCCCCT |
| SHOX_CpG1_Mlu_F | CTCACGCGTAGAAGGGGAGAAACGTCACC |
| SHOX_CpG1_XhoI_R | CAGCTCGAGAGTTCCAAAACCGAGCTCAC |
| SHOX_CpG2_KpnI_2F | GACGGTACCGCTGTGCCATCTCACACAAG |
| SHOX_CpG2_XhoI_R | CAGCTCGAGAAGACATCTCAGGCCCTACG |
| SHOX_CpG2_part1 XhoI_R | CAGCTCGAGCCATTTTTGGAAGGGAGAGC |
| SHOX_CpG2_part2_KpnI_F | GACGGTACCCCCTTCCAAAAATGGGATCT |
| SHOX_CpG2_+131_XhoI_R | CAGCTCGAGGCCAGTCCGCTCTCCAAAAC |
| SHOX_CpG2_+109_Kpn_F | GACGGTACCAAGTTTTGGAGAGCGGACTG |
|  |  |
| **Primers for qRT-PCR** | |
| **Name** | **Sequence (5'® 3')** |
| SHOXfor | CCTACGTCAACATGGGAGCCTTAC |
| SHOXrev | CCCGAAGGGCGGCGGG |
| HoxA9TaqmanFor | AAAACAATGCTGAGAATGAGAGC |
| HoxA9TaqmanRev | TATAGGGGCACCGCTTTTT |
| HOXD9TaqmanFor | AGCAGCAACTTGACCCAAA |
| HOXD9TaqmanRev | CGGGTGAGGTACATGTTGAA |
| HOXD10TaqmanFor | AGTGCAGGAGAAGGAAAGCA |
| HOXD10TaqmanRev | TCGCGGGTGAGGTACATATT |
| Pbx1TaqmanFor | ACCATACGGAGCTGGAGAAA |
| Pbx1TaqmanRev | GGGTAAGGGTTGCTGAGATG |
| Meis1TaqmanFor | TCACACTGGCCTTAAAGAGGA |
| Meis1TaqmanRev | CCGTAATGGGGTAGATCGTC |
| PPIAfor | CGGGAGGCCAGGCTCGT |
| PPIArev | TGAAAGCAGGAACCCTTATAACCAA |
| SDHAfor | TGGGAACAAGAGGGCATCTG |
| SDHArev | CCACCACTGCATCAAATTCATG |
|  | |
| **DNA oligos used in EMSA experiments** | |
| **Name** | **Sequence (5'® 3')** |
| SHOX_CpG2_Ol1_F | GGGGATCTTTCCCCCTTCGCACCAAGGTGTACGGACGCCAAACAGTGATGAAATGAGAAGAAAGCCAATTGCCGGCCT |
| SHOX_CpG2_Ol1_R | GGGAGGCCGGCAATTGGCTTTCTTCTCATTTCATCACTGTTTGGCGTCCGTACACCTTGGTGCGAAGGGGGAAAGATC |
| SHOX_CpG2_Ol2_F | GGGGTCTCTGCGTGCGTCCGCCGCGGAGCCCGGAGACCAGTAATTGCACCAGACAGGCAGCGCATGGGGGGCTGGGCGA |
| SHOX_CpG2_Ol2_R | GGGTCGCCCAGCCCCCCATGCGCTGCCTGTCTGGTGCAATTACTGGTCTCCGGGCTCCGCGGCGGACGCACGCAGAGAC |
| SHOX_CpG2_Ol3_F | GGGTCGCCGCGTATAAATAGTGAGATTTCCAATGGAAAGGCGTAAATAACAGCGCTGGTGATCCACCCGCGCGCACGGG |
| SHOX_CpG2_Ol3_R | GGGCCCGTGCGCGCGGGTGGATCACCAGCGCTGTTATTTACGCCTTTCCATTGGAAATCTCACTATTTATACGCGGCGA |
| CpG2_Oligo2a_F | GGGGTCTCTGCGTGCGTCCGCCGCGGAGCCCGGA |
| CpG2_Oligo2a_R | GGGTCCGGGCTCCGCGGCGGACGCACGCAGAGAC |
| CpG2_Oligo2b_F | GGGGCCCGGAGACCAGTAATTGCACCAGACAGGC |
| CpG2_Oligo2b_R | GGGGCCTGTCTGGTGCAATTACTGGTCTCCGGGC |
| CpG2_Oligo2c_F | GGGCCAGACAGGCAGCGCATGGGGGGCTGGGCGA |
| CpG2_Oligo2c_R | GGGTCGCCCAGCCCCCCATGCGCTGCCTGTCTGG |
| CpG2_Oligo3a_F | GGGTCGCCGCGTATAAATAGTGAGATTTCCAATG |
| CpG2_Oligo3a_R | GGGCATTGGAAATCTCACTATTTATACGCGGCGA |
| CpG2_Oligo3b_F | GGGTTCCAATGGAAAGGCGTAAATAACAGCGCTG |
| CpG2_Oligo3b_R | GGGCAGCGCTGTTATTTACGCCTTTCCATTGGAA |
| CpG2_Oligo3c_F | GGGACAGCGCTGGTGATCCACCCGCGCGCACGGG |
| CpG2_Oligo3c_R | GGGCCCGTGCGCGCGGGTGGATCACCAGCGCTGT |
| CpG2_Oligo2bMut_F | GGGGCCCGGAGACCAGGCCGGGCACCAGACAGGC |
| CpG2_Oligo2bMut_Rev: | GGGGCCTGTCTGGTGCCCGGCCTGGTCTCCGGGC |
| CpG2_Oligo3bMut_F | GGGTTCCAATGGAAAGGCGGCCCGAACAGCGCTG |
| CpG2_Oligo3bMut_R | GGGCAGCGCTGTTCGGGCCGCCTTTCCATTGGAA |
| Ch_oligo2b_F | **GGG**CGGAGCGGGCGGTAATTGCTGCAGACGGGG |
| Ch_oligo2b_R | **GGG**CCCCGTCTGCAGCAATTACCGCCCGCTCCG |
| Ch_oligo3b_F | **GGG**CTCAAATTGAAAGGCATAAATAACAGCGGGA |
| Ch_oligo3b_R | **GGG**TCCCGCTGTTATTTATGCCTTTCAATTTGAG |
| Ch_oligo2bMut_F | **GGG**CGGAGCGGGCGG**GCCGG**GCTGCAGACGGGG |
| Ch_oligo2bMut_R | **GGG**CCCCGTCTGCAGCCCGGCCCGCCCGCTCCG |
| Ch_oligo3bMut_F | **GGG**CTCAAATTGAAAGGCA**GCCCG**AACAGCGGGA |
| Ch_oligo3bMut_R | **GGG**TCCCGCTGTTCGGGCTGCCTTTCAATTTGAG |
|  | |

| **Primers used for Chromatin immunoprecipitation** | |
| --- | --- |
| ChIP_HOXA9_contr1_F | GGAGAGCAAAGTGTCCTCCA |
| ChIP_HOXA9_contr1_R | GGGAAAATGTAACGGGGACT |
| ChIP_HOXA9_contr2_F | CAGTGAGCAGCTGTGAGAGG |
| ChIP_HOXA9_contr2_R | AGCAACTTTCTGGGTCCTCA |
| ChIP HOXA9 Amp1_F | GACGGTACCCCCTTCCAAAAATGGGATCT |
| ChIP HOXA9 Amp1_R | CTGCCTGTCTGGTGCAATTA |
| ChIP HOXA9 Amp2_F | CGCCAAACAGTGATGAAATG |
| ChIP HOXA9 Amp2_R | ACCAGCGCTGTTATTTACGC |
|  |  |

| **Primers for cloning of the expression constructs** | |
| --- | --- |
| **Name** | **Sequence (5’® 3’)** |
| HoxA9_HindIII_For | CGCAAGCTTACGGTGATGGCCACCACTG |
| HoxA9_BamHI_Flag_Rev | CGCGGGATCCTCACTTGTCATCGTCGTCCTTGTAGTCCTCGTCTTTTGCTCGGTCT |
| HOXA10_HindIII_2For | CGCAAGCTTGATTTCGGAAATGTGTCAAGG |
| HOXA10_EcoRI_Flag_Rev | GCGGAATTCTCACTTGTCATCGTCGTCCTTGTAGTCGGAAAAATTAAAGTTGGCTGTGAG |
| HOXD9_HindIII_For | CGCAAGCTTGCCTCAAAATGTCTTCCAGTG |
| HOXD9_XhoI_Flag_Rev | GTGCTCGAGTCACTTGTCATCGTCGTCCTTGTAGTCGTCTCCTTTGGGGCATTTCTC |
| HOXD10_HindIII_For | CGCAAGCTTCTTCCCCAAAATGTCCTTTCC |
| HOXD10_EcoRI_Flag_Rev | CGCGAATTCTCACTTGTCATCGTCGTCCTTGTAGTCAGAAAACGTGAGGTTGGCGG |
| Meis1_HindIII_For | CGCAAGCTTGGCCGATGGCGCAAAGGTAC |
| Meis1_BamHI_Flag_Rev | CGCGGGATCCTCACTTGTCATCGTCGTCCTTGTAGTCCATGTAGTGCCACTGCCCCT |
| Pbx1_HindII_For | CGCAAGCTTTTGGAGATGGACGAGCAGCC |
| Pbx1_BamHI_Flag_Rev | CGCGGGATCCTCACTTGTCATCGTCGTCCTTGTAGTCGTTGGAGGTATCAGAGTGAA |
|  | |
| **Mutagenesis primers** | |
| **Name** | **Sequence (5'® 3')** |
| HOXA9QuikChangeR257/58 | CAAGATCTGGTTCCAGCCCGGGATGAAAATGAAGAAAATC |
| HOXA9QuikChangeK223 | GACCCTGGAACTGGAGGAAGAGTTTCTGTTCAACATG |
| HOXD9 K292 > E292 | Ccagacgcttgagctggaggaagaattcctcttcaacatg |
|  |  |
| HOXD9 R326 > P326 R327> G327 | Aaaatctggtttcagaaccctgggatgaaaatgaaaaagatg |
|  | |
| **Primers for cloning of HOXA9-GST** | |
| **Name** | **Sequence (5'® 3')** |
| HOXA9pGEXEcoRI_F | CGCGAATTCACGGTGATGGCCACCACTG |
| HOXA9pGEXXhoI_R | GCGCTCGAGTCACTCGTCTTTTGCTCGGTC |
| chHOXA9pGEXEcoRI_F | gcgcGAATTCatgtcggcccccggga |
| chHOXA9pGEXNotI_R | gcgcCGCCGGCGtcattcgtccttcgctcggtct |
|  | |
| **Primers for cloning of Hoxa9-RCAS** | |
| **Name** | **Sequence (5'® 3')** |
| cHoxa9 SLAX13SpeI_For | GACGTCTCCCATGTCGGCCCCCGGGA |
| cHoxa9 SLAX13SpeI_Rev | GACGTCTCGAATTCTCATTCGTCCTTCGCTCGGTCT |
|  | |
| **Primers for cloning of in situ hybridization probes** | |
| **Name** | **Sequence (5'® 3')** |
| chiSHOX_1_For | GAGCTTGGGAACTCCGATT |
| chiSHOX_2_Rev | TTCAGACAGTCCCAGCCTCT |
| chiHOXA9_For | TACGTGGACTCCTTCCTGGT |
| chiHOXA9_Rev | TCTCTCCGTTTGCCTCACTT |

Supplementary Table 1
